# Vascular Function in Patients After Myocardial Infarction: The Importance of Physical Activity

**DOI:** 10.3389/fphys.2021.763043

**Published:** 2021-12-24

**Authors:** Jure Tršan, Daniel Košuta, Uroš Rajkovič﻿﻿﻿, Zlatko Fras, Borut Jug, Marko Novaković﻿﻿﻿

**Affiliations:** ^1^Division of Internal Medicine, Department of Vascular Diseases, University Medical Centre Ljubljana, Ljubljana, Slovenia; ^2^Faculty of Medicine, University of Ljubljana, Ljubljana, Slovenia; ^3^Faculty of Organizational Sciences, University of Maribor, Kranj, Slovenia

**Keywords:** physical activity, myocardial infarction, endothelial function, arterial stiffness, cardiovascular risk factors

## Abstract

**Background:** Patients after myocardial infarction have impaired vascular function. However, effects of lifestyle, e.g., physical activity level, on endothelial function and arterial stiffness remain scarce. The aim of our study was to investigate effects of physical activity level and risk factors on endothelial function and arterial stiffness.

**Methods:** In this cross-sectional study, we ultrasonographically assessed parameters of vascular function, namely flow mediated dilation (FMD) of the brachial artery and carotid artery stiffness in patients after myocardial infarction referred to the cardiac rehabilitation. The International Physical Activity Questionnaire (IPAQ) was obtained from all participants. Based on the IPAQ, patients were classified into three groups: vigorous, moderate, and low physical activity engagement. ANOVA was used for comparison among three groups using Bonferroni correction to determine differences between two sub-groups.

**Results:** One hundred and eight patients after myocardial infarction (mean age 53 ± 10 years) were included. There were significant differences in terms of FMD (8.2 vs. 4.2 vs. 1.9%, *p* < 0.001) and pulse wave velocity (PWV), a measure of arterial stiffness (6.1 vs. 6.4 vs. 6.9 m/s, *p* = 0.004) among groups of vigorous, moderate, and low physical activity engagement, respectively. However, in younger patients only FMD remained associated with physical activity level, while arterial stiffness was not. Low physical activity engagement was a significant predictor of both FMD and PWV in univariate and multivariate models, adjusted for age, sex, and other risk factors.

**Conclusion:** Low physical activity level is associated with impaired endothelial function and increased arterial stiffness in patients after myocardial infarction. Future studies are warranted to address this issue in a context of cardiac rehabilitation protocols optimization in order to improve vascular function in these patients.

## Introduction

Myocardial infarction is a terminal step in the atherosclerotic pathophysiology. It starts with the functional impairment (Poredoš, 2002; Davignon and Ganz, 2004) and leads to the structural changes, mostly in the coronary, carotid, and arteries of the lower extremities (Kaspar et al., 2019). The ability of the vascular endothelium and smooth muscle to release the molecules responsible for maintaining relaxed vascular tone, particularly nitric oxide, is impaired, resulting in endothelial dysfunction (Hadi et al., 2005). With impaired endothelial function, vasoconstrictive, proinflammatory, proliferative, and thrombogenic factors predominate, creating a markedly proatherogenic state (Chistiakov et al., 2015). Endothelial dysfunction has been associated with atherosclerotic plaque formation, progression, and increased vulnerability (Sitia et al., 2010). Therefore, patients with established coronary artery disease, especially after myocardial infarction, in most cases already have impaired endothelial function, often determined as flow-mediated dilation (Erzen et al., 2007). Arterial stiffness, on the other hand, is a measure of the elastic properties of the arteries and relative contribution of collagen and elastin. It is consisted of the before mentioned functional changes, related to the flow mediated dilation (FMD), but also of the structural changes, as a consequence of the extracellular matrix degeneration, collagen deposition, and elastin fragmentation, and finally to the diminished arterial elastic properties (10; Janić et al., 2014). Studies have shown that oxidative stress and inflammation are the main causes of vascular stiffening (Ashor et al., 2014).

However, certain lifestyle changes can modify vascular function. Physical activity is recommended by the relevant guidelines for healthy individuals, various conditions including cardiovascular patients (Authors/Task Force Members et al., 2016; Pelliccia et al., 2020), as it is, among other benefits, associated with both endothelial function and arterial stiffness improvement (Di Francescomarino et al., 2009; Bernardi et al., 2018; Alves et al., 2019). Also, it improves other cardiovascular risk factors, such as obesity, arterial hypertension, dyslipidaemia, or diabetes mellitus (Novakovic et al., 2017; Winzer et al., 2018). Vigorous physical activity additionally upgrades vascular function (Coovert et al., 2018) as increased shear stress additionally enhances nitric oxide secretion (Cunningham and Gotlieb, 2005). Furthermore, the extent of the age-related reduction in central arterial compliance is attenuated in those who regularly perform endurance exercise (Tanaka et al., 2000; Boreham et al., 2004; Zieman et al., 2005; Lavie et al., 2015) and a relatively brief period of regular aerobic exercise can restore some of the loss of central arterial compliance (Tanaka et al., 2000; Lavie et al., 2015). The beneficial effects of chronic aerobic exercise on arterial stiffness are associated with increased pulsatile flow and stretch and consequent enhanced nitric oxide bioavailability (6). Protection against systemic oxidative stress and inflammation induced by physical activity is posited to be a primary mechanism for the observed reductions in arterial stiffness (Lavie et al., 2015). Regular physical activity increases the production of anti-inflammatory cytokines (such as interleukin 4 and 10) and reducing proinflammatory cytokines (such as interleukin 6 and tumor necrosis factor alpha; Teixeira-Lemos et al., 2011; Ashor et al., 2014). Nevertheless, little is known how these changes due to regular physical activity are affected by age, especially in individuals with established atherosclerotic disease, e.g., after myocardial infarction.

In the present study, we thought to compare vascular function (endothelial function and arterial stiffness) in terms of physical activity level, in patients after myocardial infarction. Additionally, our aim was to check if there are differences in these associations in younger and older patients.

## Materials and Methods

### Study Population and Design

Patients older than 18 years of age, who suffered myocardial infarction in the period less than 4 months, were screened for the study before commencement of the outpatient clinic cardiac rehabilitation program at the Centre for Preventive Cardiology, Department of Vascular Diseases, University Medical Centre Ljubljana, Slovenia. Exclusion criteria included acute illness 1 month prior to inclusion, non-cardiovascular diseases (CVDs) decompensation requiring hospital admission, emergency or unplanned specialist management, unstable dysrhythmias, pregnancy, and intellectual development disorder. The study was approved by the National Medical Ethics Committee and has been performed in accordance with the Declaration of Helsinki and its later amendments. Written consent was obtained from all patients prior to their inclusion.

Patients’ clinical data was analyzed, vascular function, and cardiopulmonary exercise testing were performed.

### Cardiopulmonary Exercise Testing

Maximal cardiopulmonary exercise testing (CPET) was performed using cycle ergometer Schiller CS-200. CPET protocol consisted of initial 3-min interval without workload, which was followed by gradual increase in workload every minute by one tenth of maximal estimated workload, calculated based on age, gender, and height.

The flow of oxygen and carbon dioxide during exercise was monitored with a mouthpiece connected to the device, ECG and heart rate (HR) were monitored continuously during and immediately after the CPET and blood pressure was measured manually every 2 min. Patients gave their maximal effort before they stopped cycling. Testing was discontinued at the onset of symptoms (exhaustion, dyspnea, and chest or leg pain), signs (increased blood pressure to more than 250/120 mmHg, decreased systolic blood pressure by more than 10 mmHg, peak HR, and decreased heart rate), and/or ischemic changes in the electrocardiographic record or occurrence of arrhythmias.

Data obtained from the CPET were, as follows: peak oxygen uptake (VO_2_peak), resting and peak heart rate, resting and peak systolic and diastolic blood pressure, and peak systolic pressure times peak heart rate.

### Vascular Function

Endothelial function was determined by endothelium-dependent vasodilation (FMD), using Aloka ProSound α7 ultrasound device and a 10 MHz linear array transducer. The probe was positioned with a dedicated mechanical support. If this was not possible, we performed the measurements manually. The evaluations were made by two skilled observers with previous data on intra- and interobserver variability for FMD and pulse wave velocity (PWV) measurements. For this study, we have performed additional intra- and inter-observer reliability appraisal. For FMD, intra- and inter-rater reliability coefficients were 0.906 and 0.821, respectively and for PWV, intra- and inter-rater reliability coefficients were 0.976 and 0.969, respectively. Firstly, the right brachial artery was scanned in the longitudinal section approximately 5 cm above the antecubital fossa to find the clearest image of the anterior and posterior arterial wall layer. The first image was then obtained. Secondly, to increase hyperemic flow, the cuff was placed and inflated on the middle third of the forearm to a pressure of 50 mmHg above the patient’s systolic pressure for 4.5 min. About 60 s after cuff deflation, the second image was obtained. At the post processing, at least three measurements of the arterial diameter were obtained, and the average value of measurements was determined at baseline (d1) and 60 s after the cuff deflation (d2). FMD was expressed as a percentage change of the diameter after reactive hyperemia relative to the baseline scan using the following formula: (d2-d1)/d1 and expressed in percentage.

The endothelium-independent vasodilation test was not performed due to the logistic reasons. The endothelium-independent dilation test is performed using the nitroglycerin. As nitroglycerin is known to affect exercise performance, we decided not to perform it as participants underwent cardiopulmonary exercise testing or had the first cardiac rehabilitation session just after the ultrasound examination.

Arterial stiffness was determined by measuring the parameters of carotid arterial stiffness, e.g., β stiffness coefficient and PWV. The measurements were performed using Aloka ProSound α7 ultrasound device, with echo tracking software to determine carotid stiffness parameters through the analysis of the pulse waves, and a 10 MHz linear array transducer. Patients rested in the supine position for 10 min before hemodynamic measurements. Blood pressure was measured on the left upper extremity and arterial stiffness measurements were performed on the right common carotid artery (RCCA). Patients had their head elevated at around 45° and tilted to the left by 30°.

We initially scanned the RCCA about 2 cm before the carotid bifurcation. Then the cursor pair was positioned on the anterior and posterior walls of the RCCA artery. The dedicated program then analyzed the waveform signals caused by periodic changes in the diameter of the common carotid artery based on the systolic and diastolic pressures and automatically determined the stiffness coefficient β and PWV. Calibration of the blood pressure was performed twice and for each we performed six consecutive measurements. The stiffness coefficient β and PWV were determined as average value of 12 measurements (Novaković et al., 2017).

### Self-Estimated Physical Activity Level

In order to determine physical activity level before myocardial infarction, the International Physical Activity Questionnaire (IPAQ) was determined twice within 2 weeks in order to prevent possible overestimation and obtain reliable physical activity level. According to the authors’ guidelines, physical activity level was classified into three levels, as follows: vigorous, moderate, and low physical activity level (Craig et al., 2003).

### Statistical Analysis

The normal distribution of variables was assessed with the Kolmogorov-Smirnov test after previous graphical description. Normally distributed continuous variables were expressed as mean values and SDs, while asymmetrically distributed continuous variables were expressed as median and interquartile ranges. Categorical variables were expressed as numbers and percentages. ANOVA with Bonferroni adjustment, and Kruskal-Wallis test were used for comparison of three groups for normally and asymmetrically distributed variables, respectively. Independent samples *t*-test and Mann-Whitney U tests were performed for comparison of two groups for normally and asymmetrically distributed variables, respectively. Univariate and multivariate linear regression analyses were performed to determine predictors of the vascular function parameters. Chi square test was used to assess differences of categorical variables. Data were analyzed with the IBM SPSS Statistics v. 20. A value of *p* < 0.05 was considered statistically significant.

## Results

There were 108 patients in our study, 21 of them (19.4%) were females, average age was 55.4 years (Table 1). Median time from myocardial infarction was 85 days. According to the IPAQ questionnaire, patients were divided into three groups: vigorous, moderate, and low physical activity engagement (Table 1). There were significant differences among groups in terms of PWV (6.1 vs. 6.4 vs. 6.9 m/s, *p* = 0.004) and FMD (8.2 vs. 4.2 vs. 1.9%, *p* < 0.001) among vigorous, moderate, and low physical activity level group, respectively. Also, differences were significant among groups in terms of exercise capacity (24.4 vs. 22.9 vs. 19.0 ml/kg/min, *p* = 0.001; Table 1).

**Table 1:** Baseline characteristics.

	Vigorous physical activity (*n* = 26)	Moderate physical activity (*n* = 56)	Low physical activity (*n* = 26)	Significance
Age, mean (SD), years	52.9 (9.6)	56.0 (10.8)	56.6 (9.2)	0.351
Female gender, n (%)	4 (15)	12 (21)	5 (19)	0.813
BMI, mean (SD), kg/m^2^	28.0 (3.6)	28.9 (4.7)	30.3 (4.9)	0.192
Days from myocardial infarction, median (Q1–Q3)	88 (67–105)	80 (67–108)	89 (77–113)	0.699
STEMI, n (%)	17 (65)	30 (54)	19 (73)	0.212
PCI LAD, n (%)	13 (50)	30 (54)	11 (42)	0.637
Preserved LV EF, n (%)	18 (69.2)	44 (78.6)	17 (65.4)	0.399
Regional contraction disturbances, n (%)	21 (80.8)	37 (66.1)	21 (80.8)	0.227
Arterial hypertension, n (%)	15 (58)	43 (77)	21 (81)	0.116
Dyslipidaemia, n (%)	14 (54)	40 (71)	14 (54)	0.167
Diabetes mellitus, n (%)	1 (4)	6 (11)	1 (4)	0.396
Family history of CVD, n (%)	8 (31)	23 (41)	13 (50)	0.368
Smoker, n (%)	11 (42)	22 (39)	15 (58)	0.287
Resting HR, mean (SD), min(−1)	59 (7)	61 (9)	58 (6)	0.293
Peak HR, mean (SD), min(−1)	135 (13)	130 (17)	124 (15)	0.072
Resting mean pressure, mean (SD), mmHg	94 (12)	93 (10)	96 (13)	0.575
Peak mean pressure, mean (SD), mmHg	126 (13)	121 (16)	121 (15)	0.323
Peak systolic pressure peak HR, mean (SD)	26,207 (4322)	24,447 (4923)	23,581 (5814)	0.157
VO_2_peak, mean (SD), ml/kg/min	24.2 (5.3)	22.7 (5.9)	19.1 (5.3)	0.004^1^
PWV, mean (SD), m/s	6.1 (0.8)	6.4 (0.8)	6.9 (1.0)	0.004^1^
Beta stiffness, mean (SD)	7.8 (2.6)	8.4 (2.1)	9.7 (2.7)	0.013^2^
FMD, mean (SD), %	8.2 (4.7)	4.2 (3.9)	1.9 (5.1)	<0.001^3^

1 *vigorous vs. low, moderate vs. low physical activity*.

2 *vigorous vs. low physical activity*.

3 *vigorous vs. moderate, vigorous vs. low physical activity*.

*BMI, body mass index; Q1–Q3. interquartile range; STEMI, ST segment elevation myocardial infarction; PCI, percutaneous coronary intervention; LAD, left anterior descending artery; LV EF, left ventricular ejection fraction; CVD, cardiovascular disease; HR, heart rate; VO_2_peak, peak rate of oxygen consumption; PWV, pulse wave velocity; and FMD, flow-mediated dilation*.

**Figure 1:**
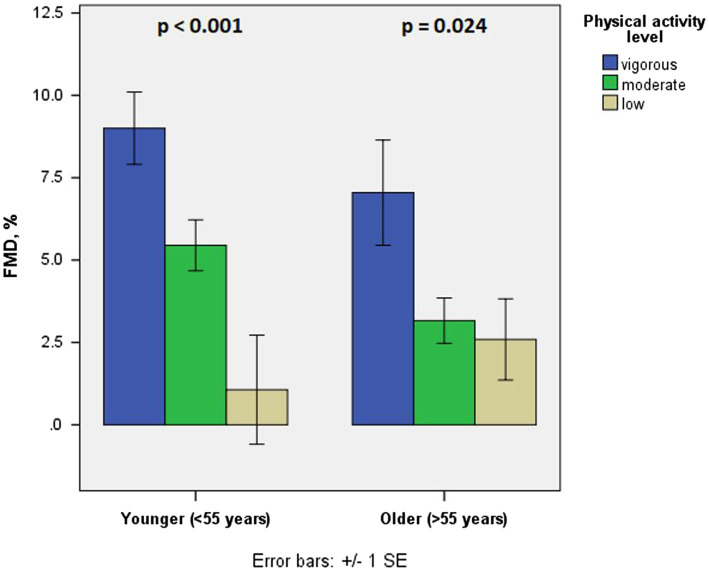
Flow-mediated dilation in different age groups in terms of physical activity level.

**Figure 2:**
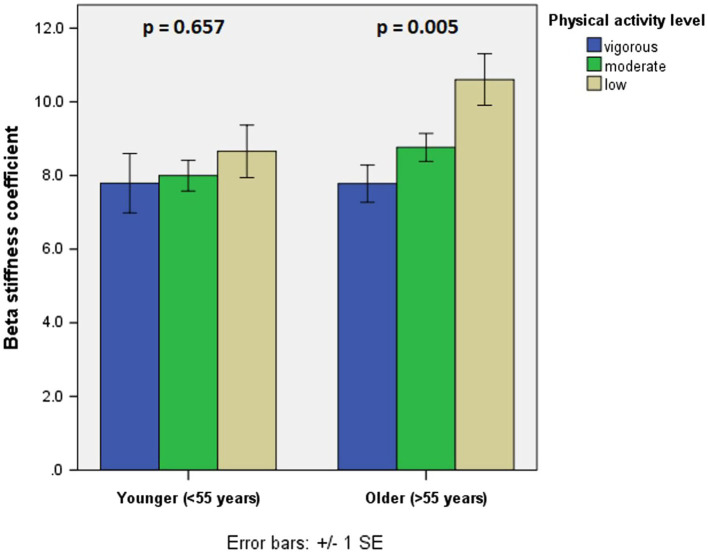
Beta stiffness coefficient in different age groups in terms of physical activity level.

We have additionally divided our sample into two subgroups based of median (<55 years vs. ≥55 years of age; Table 2). In younger (<55 years) patients, differences among vigorous, moderate, and low physical activity groups in terms of PWV and beta stiffness coefficient were not significant (6.0 vs. 6.3 vs. 6.5 m/s, *p* = 0.353 and 7.8 vs. 8.0 vs. 8.6, *p* = 0.657, respectively). However, there were significant differences in terms of FMD (9.0 vs. 5.4 vs. 1.1%, *p* < 0.001). Conversely, in older half of patients (≥55 years of age), differences in terms of both arterial stiffness parameters and FMD were significant (6.2 vs. 6.6 vs. 7.3 m/s, *p* = 0.009 for PWV, 7.8 vs. 8.8 vs. 10.6, *p* = 0.005 for beta stiffness coefficient, and 7.0 vs. 3.2 vs. 2.6%, *p* = 0.024 for FMD; Figures 1, 2).

**Table 2:** Baseline characteristics according to age.

	Younger (<55 years; *n* = 53)	Older (≥55 years; *n* = 55)	Significance
Female gender, n (%)	10 (19)	11 (20)	0.882
BMI, mean (SD), kg/m^2^	29.4 (4.5)	28.6 (4.7)	0.344
Days from myocardial infarction, median (Q1–Q3)	90 (70–112)	84 (67–102)	0.271
STEMI, n (%)	35 (66)	31 (56)	0.303
PCI LAD, n (%)	32 (60)	22 (40)	0.034
Preserved LV EF, n (%)	39 (74)	40 (73)	0.920
Regional contraction disturbances, n (%)	41 (77)	38 (69)	0.332
Arterial hypertension, n (%)	36 (68)	43 (78)	0.229
Dyslipidaemia, n (%)	31 (58)	37 (67)	0.345
Diabetes mellitus, n (%)	4 (7)	4 (7)	0.957
Family history of CVD, n (%)	27 (51)	17 (31)	0.034
Smoker, n (%)	30 (57)	18 (33)	0.013
VO_2_peak, mean (SD), ml/kg/min	23.2 (6.4)	21.2 (5.1)	0.082
Beta stiffness, mean (SD)	8.1 (2.5)	9.0 (2.3)	0.044
PWV, mean (SD), m/s	6.3 (0.9)	6.7 (0.9)	0.018
FMD, mean (SD), %	5.5 (5.2)	3.8 (4.5)	0.079
Vigorous physical activity, n (%)	15 (28)	11 (20)	0.601
Moderate physical activity, n (%)	26 (49)	30 (54)	
Low physical activity, n (%)	12 (23)	14 (25)	

*BMI, body mass index; Q1–Q3, interquartile range; STEMI, ST segment elevation myocardial infarction; PCI, percutaneous coronary intervention; LAD, left anterior descending artery; LV EF, left ventricular ejection fraction; CVD, cardiovascular disease; HR, heart rate; VO_2_peak, peak rate of oxygen consumption; PWV, pulse wave velocity; and FMD, flow-mediated dilation*.

Furthermore, we have performed univariate and multivariate linear regression analyses using PWV and FMD as dependent variables (Table 3). In terms of PWV, age was shown to be an independent predictor in univariate analysis, while mean blood pressure and low level of physical activity emerged as independent predictors in both univariate and multivariate analyses. As for FMD, low level of physical activity and dyslipidaemia appeared to be independent predictors in both univariate and multivariate linear regression analysis.

**Table 3:** Linear regression models with PWV and FMD as dependent variables.

	Pulse wave velocity					
	Univariate			Multivariate		
	*B*	*β*	CI	*B*	*β*	CI
Age ≥ 55 years	0.416	0.227	0.072–0.761	0.318	0.173	−0.030–0.665
Male gender	−0.093	−0.040	−0.540–0.353	−0.164	−0.716	−0.617–0.290
Mean arterial pressure	0.016	0.191	0.000–0.031	0.017	0.212	0.002–0.032
Low physical activity	0.578	0.272	0.185–0.971	0.524	0.203	0.122–0.925
Arterial hypertension	0.289	0.139	−0.107–0.684	0.275	0.206	−0.134–0.684
Dyslipidaemia	0.099	0.052	−0.267–0.464	0.095	0.183	−0.268–0.458
Diabetes mellitus	−0.473	−0.135	−1.142–0.197	−0.394	−0.112	−1.040–0.253
Cigarette smoking	−0.174	−0.094	−0.528–0.181	−0.202	−0.109	−0.561–0.157
Obesity	0.071	0.057	−0.169–0.311	0.041	0.033	−0.202–0.284
Flow-mediated dilation						
	Univariate			Multivariate		
	*B*	*β*	CI	*B*	*β*	CI
Age ≥ 55 years	−1.670	−0.170	−3.538–0.199	−1.329	−0.135	−3.209–0.551
Male gender	−0.597	−0.048	−2.990–1.796	−0.323	−0.026	−2.774–2.127
Mean arterial pressure	−0.023	−0.052	−0.106–0.061	−0.019	−0.044	−0.099–0.061
Low physical activity	−3.351	−0.295	−5.443–(−1.259)	−3.107	−0.273	−5.280–(−0.935)
Arterial hypertension	−0.962	−0.087	−3.093–1.169	−1.102	−0.099	−3.312–1.107
Dyslipidaemia	−2.355	−0.231	−4.265–(−0.445)	−2.282	−0.224	−4.242–(−0.322)
Diabetes mellitus	0.014	0.001	−3.606–3.633	−0.416	−0.022	−3.911–3.078
Cigarette smoking	1.306	0.132	−0.585–3.197	1.203	0.121	−0.737–3.144
Obesity	−0.171	−0.026	−1.459–1.116	−0.236	−0.035	−1.550–1.078

## Discussion

We have shown that lower physical activity engagement is associated with more diminished vascular function, namely increased arterial stiffness and impaired endothelial function. However, while both associations have been confirmed in older patients after myocardial infarction, in younger patients only FMD seems to be significantly associated with the level of physical activity engagement, while arterial stiffness does not.

To our knowledge, this is the first study to analyze effects of physical activity level and other risk factors on arterial stiffness and endothelial function in patients shortly after a myocardial infarction. Our study has shown that lower level of physical activity levels is independently associated with increased arterial stiffness after adjustment for age, gender, mean blood pressure, and other cardiovascular risk factors.

Recent literature reports are in line with our results regarding association between arterial stiffness and aerobic capacity (Alves et al., 2019). Exercise capacity is a widely used physiological variable, dependent on level of various factors, and is mainly a consequence (rather than a cause) of physical activity. Although there are strict definitions and differences between physical activity and exercise, it seems that aerobic bodily movement with all its physiological benefits (increased shear stress, modulating pro- and antioxidative cytokines, beneficial effects on cardiovascular risk factors; Novakovic et al., 2017) eventually lead to decrease of arterial stiffness, which is shown in our study and confirmed in reviews and meta-analyses (Ashor et al., 2014; Oliveira et al., 2014).

We have also confirmed significant associations between levels of physical activity and endothelial function. Most studies have shown significant beneficial effects of more intensive bodily movement (including exercise) on endothelial function in healthy populations and various cardiovascular conditions (Pahkala et al., 2008; Payvandi et al., 2009; Winzer et al., 2018). More vigorous physical activity leads to increased local and systemic blood flow, increases shear stress and secretion of nitric oxide, which all cause chronic flow-mediated dilation improvement (Winzer et al., 2018). Patients after myocardial infarction have significantly lower FMD, as compared to their healthy peers (Erzen et al., 2007), but it seems that the decrease can be at least lowered (or maybe improve and even increase) with more vigorous physical activity.

Another intriguing result of our study are discrepancies between associations of physical activity level and arterial stiffness in different age groups. According to our results, only in older patients after myocardial infarction, arterial stiffness is associated with physical activity level, while in younger it does not, which is in line with results of Tanaka et al. (2000), examining healthy individuals. There are a few possible explanations for such a result. Firstly, in younger patients there may be a dominance of structural aspects of arterial stiffness, which are less prone to lifestyle changes including physical activity. On the contrary, in older coronary artery disease patients, chronic effects of regular physical activity, together with ageing, which was shown to be another significant predictor of arterial stiffness according to our results, may lead to more predictable arterial stiffness. These changes in older patients may not be dependent on structural changes in collagen and elastin, but of modifications of gene expression associated with local vasodilatory signaling (Maeda et al., 2005; Jakovljevic, 2018). Conversely, effects of physical activity on dynamic aspects of vascular function (e.g., FMD) seem to be significant in both age categories. These effects of physical activity (and exercise) on endothelial function in different age categories was confirmed in most studies and are in line with our results (Pahkala et al., 2008; Payvandi et al., 2009; Winzer et al., 2018).

Tobacco smoking contributes greatly to initiation of atherogenesis and consequent early myocardial infarction (Ambrose and Barua, 2004). The most prominent processes are promotion of endothelial dysfunction and inflammation in vascular wall, oxidation of proatherogenic lipids and decrease of high-density lipoprotein. Cigarette smoke also activates platelets, stimulates the coagulation cascade, and reduces fibrinolysis, thus inducing markedly procoagulant state (Messner and Bernhard, 2014). Not only is smoking responsible for a significant proportion of myocardial infarctions in young adults but it was also shown that increase in cardiovascular risk caused by smoking is greater in the young than in the old, for men and women (Teo et al., 2006).

Mean arterial pressure was shown to be another significant predictor of the arterial stiffness. This significant association provides a key understanding of the arterial elasticity and consequences of the arterial wall with changes in arterial pressure. Higher blood pressure decreases arterial extensibility mostly through the engagement of the collagen fibers in the media, which are dependent of higher blood pressure, so the wall becomes stiffer, thus quicker conducting pulse waves. On the contrary, lower blood pressure mostly engages elastin component of the arterial media, providing arterial distensibility (Janić et al., 2014). Although pulse wave velocity, as the most common marker of arterial stiffness, is often highly correlated with blood pressure, it additionally emerged as an independent predictor of worse cardiovascular outcomes, independently of blood pressure (Mitchell et al., 1997; Laurent et al., 2001).

Dyslipidaemia as a risk factor emerged as a significant factor of endothelial dysfunction (Steinberg et al., 1997). As already mentioned, endothelial function is an initial step in the atherosclerotic pathway (Poredoš, 2002; Davignon and Ganz, 2004), among other factors caused by higher levels of lipoproteins with low density (Borén et al., 2020). As cholesterol particles build in atherosclerotic plaques, it seems that the whole pathophysiological cascade affects functional component of vascular function. According to our results, it seems that blood pressure more affects arterial stiffness, while dyslipidaemia is more responsible for the endothelial function impairment.

There are some limitations of our study. Firstly, a sample size and a single-center setting. Although our sample size was relatively large in comparison to previous reports, its single-center pattern may decrease generalizability to other populations. Therefore, larger multi-center studies are needed to confirm our results. Secondly, this is a cross-sectional study and can only answer the question of association, not causality. Thirdly, physical activity level was assessed using the IPAQ questionnaire and not with the modern electronic analyzers of physical activity. Nevertheless, in our study IPAQ questionnaire classification correlated well with CPET results, which is in line with some literature reports on coronary artery disease patients (Manta et al., 2021) and other CVD patient populations (Müller et al., 2017; Mediano et al., 2018). Due to its subjectivity and potential overestimation of the physical activity level, we have additionally checked physical activity level and thus decreased possibility of overestimation. Evaluation of physical activity with electronic analyzers may not be entirely representative either, because people tend to unintentionally exaggerate their physical activity during shorter measuring periods which would be applicable to our study (Ernsting et al., 2017). Other considerations against electronic devices include lack of information technology experience of some participants and financial issues to obtain these devices. After careful consideration, we decided to use the IPAQ questionnaire as it is accessible, inexpensive, and reproducible questionnaire with easy implementation and generalization potential for practical considerations. Finally, a solution suitable for our population size might lay in a simple, easy-to-use and handy mobile phone application that would automatically record physical activity level for a longer period of time and would obtain some limited parameters of physiological importance (e.g., number of steps and/or heart rate; Strath et al., 2013).

To conclude, physical activity level is associated with endothelial function in patients after myocardial infarction. Additionally, there is a significant association between physical activity level and arterial stiffness, but only in older patients after myocardial infarction.

## Data Availability Statement

The raw data supporting the conclusions of this article will be made available by the authors, without undue reservation.

## Ethics Statement

The studies involving human participants were reviewed and approved by National Medical Ethics Committee of the Republic of Slovenia. The patients/participants provided their written informed consent to participate in this study.

## Author Contributions

JT contributed to drafting the work, acquisition, analysis, and interpretation of the data for the work, and agreed to be accountable for all aspects of the work in ensuring that questions related to the accuracy or integrity of any part of the work are appropriately investigated and resolved. DK contributed to data acquisition, analysis, and interpretation, and drafting the work. UR organized the database and contributed to the data analysis and interpretation. ZF substantially contributed to the data analysis and interpretation, and manuscript revision. BJ and MN contributed to conception and design of the study, drafting the work, revising it critically for important intellectual content, and provided approval for the final manuscript. All authors contributed to the article and approved the submitted version.

## Conflict of Interest

The authors declare that the research was conducted in the absence of any commercial or financial relationships that could be construed as a potential conflict of interest.

## Publisher’s Note

All claims expressed in this article are solely those of the authors and do not necessarily represent those of their affiliated organizations, or those of the publisher, the editors and the reviewers. Any product that may be evaluated in this article, or claim that may be made by its manufacturer, is not guaranteed or endorsed by the publisher.

## References

[ref1] Alves A. J. Oliveira N. L. Lopes S. Ruescas-Nicolau M.-A. Teixeira M. Oliveira J.. (2019). Arterial stiffness is related to impaired exercise capacity in patients with coronary artery disease and history of myocardial infarction. Heart Lung Circ. 28, 1614–1621. doi: 10.1016/j.hlc.2018.08.023, PMID: 30318391

[ref2] Ambrose J. A. Barua R. S. (2004). The pathophysiology of cigarette smoking and cardiovascular disease. J. Am. Coll. Cardiol. 43, 1731–1737. doi: 10.1016/j.jacc.2003.12.047, PMID: 15145091

[ref3] Ashor A. W. Lara J. Siervo M. Celis-Morales C. Mathers J. C. (2014). Effects of exercise modalities on arterial stiffness and wave reflection: a systematic review and meta-analysis of randomized controlled trials. PLoS One 9:e110034. doi: 10.1371/journal.pone.0110034, PMID: 25333969 PMC4198209

[ref4] Authors/Task Force Members Piepoli M. F. Hoes A. W. Agewall S. Albus C. Brotons C.. (2016). 2016 European guidelines on cardiovascular disease prevention in clinical practice: the sixth joint task force of the European society of cardiology and other societies on cardiovascular disease prevention in clinical practice (constituted by representatives of 10 societies and by invited experts): developed with the special contribution of the European association for cardiovascular prevention & rehabilitation (EACPR). Eur. J. Prev. Cardiol. 23, NP1–NP96. doi: 10.1177/2047487316653709 27353126

[ref5] Bernardi E. Merlo C. Cogo A. (2018). Endothelial function in COPD is in an intermediate position between healthy subjects and coronary artery disease patients and is related to physical activity. Lung 196, 669–672. doi: 10.1007/s00408-018-0168-9, PMID: 30284026

[ref6] Boreham C. A. Ferreira I. Twisk J. W. Gallagher A. M. Savage M. J. Murray L. J. (2004). Cardiorespiratory fitness, physical activity, and arterial stiffness: the Northern Ireland young hearts project. Hypertension 44, 721–726. doi: 10.1161/01.HYP.0000144293.40699.9a, PMID: 15452034

[ref7] Borén J. Chapman M. J. Krauss R. M. Packard C. J. Bentzon J. F. Binder C. J.. (2020). Low-density lipoproteins cause atherosclerotic cardiovascular disease: pathophysiological, genetic, and therapeutic insights: a consensus statement from the European atherosclerosis society consensus panel. Eur. Heart J. 41, 2313–2330. doi: 10.1093/eurheartj/ehz962, PMID: 32052833 PMC7308544

[ref8] Chistiakov D. A. Orekhov A. N. Bobryshev Y. V. (2015). Endothelial barrier and its abnormalities in cardiovascular disease. Front. Physiol. 6:365. doi: 10.3389/fphys.2015.00365, PMID: 26696899 PMC4673665

[ref9] Coovert D. Evans L. D. Jarrett S. Lima C. Lima N. Gurovich A. N. (2018). Blood flow patterns during incremental and steady-state aerobic exercise. J. Sports Med. Phys. Fitness 58, 1537–1543. doi: 10.23736/S0022-4707.17.07142-0, PMID: 28558444

[ref10] Craig C. L. Marshall A. L. Sjöström M. Bauman A. E. Booth M. L. Ainsworth B. E.. (2003). International physical activity questionnaire: 12-country reliability and validity. Med. Sci. Sports Exerc. 35, 1381–1395. doi: 10.1249/01.MSS.0000078924.61453.FB, PMID: 12900694

[ref11] Cunningham K. S. Gotlieb A. I. (2005). The role of shear stress in the pathogenesis of atherosclerosis. Lab. Investig. 85, 9–23. doi: 10.1038/labinvest.3700215, PMID: 15568038

[ref12] Davignon J. Ganz P. (2004). Role of endothelial dysfunction in atherosclerosis. Circulation 109, 27–32. doi: 10.1161/01.CIR.0000131515.03336.f8 15198963

[ref13] Di Francescomarino S. Sciartilli A. Di Valerio V. Di Baldassarre A. Gallina S. (2009). The effect of physical exercise on endothelial function. Sports Med. 39, 797–812. doi: 10.2165/11317750-000000000-00000, PMID: 19757859

[ref14] Ernsting C. Dombrowski S. U. Oedekoven M. O’Sullivan J. L. Kanzler M. Kuhlmey A.. (2017). Using smartphones and health apps to change and manage health behaviors: a population-based survey. J. Med. Internet Res. 19:e101. doi: 10.2196/jmir.6838, PMID: 28381394 PMC5399221

[ref15] Erzen B. Sabovic M. Sebestjen M. Poredos P. (2007). Endothelial dysfunction, intima-media thickness, ankle-brachial pressure index, and pulse pressure in young post-myocardial infarction patients with various expressions of classical risk factors. Heart Vessel. 22, 215–222. doi: 10.1007/s00380-006-0958-5, PMID: 17653514

[ref16] Hadi H. A. R. Carr C. S. Al Suwaidi J. (2005). Endothelial dysfunction: cardiovascular risk factors, therapy, and outcome. Vasc. Health Risk Manag. 1, 183–198. PMID: 17319104 PMC1993955

[ref17] Jakovljevic D. G. (2018). Physical activity and cardiovascular aging: physiological and molecular insights. Exp. Gerontol. 109, 67–74. doi: 10.1016/j.exger.2017.05.016, PMID: 28546086

[ref18] Janić M. Lunder M. Sabovič M. (2014). Arterial stiffness and cardiovascular therapy. Biomed. Res. Int. 2014:621437. doi: 10.1155/2014/621437, PMID: 25170513 PMC4142148

[ref19] Kaspar M. Baumgartner I. Staub D. Drexel H. Thalhammer C. (2019). Non-invasive ultrasound-based imaging of atherosclerosis. Vasa 48, 126–133. doi: 10.1024/0301-1526/a000747, PMID: 30324866

[ref20] Laurent S. Boutouyrie P. Asmar R. Gautier I. Laloux B. Guize L.. (2001). Aortic stiffness is an independent predictor of all-cause and cardiovascular mortality in hypertensive patients. Hypertension 37, 1236–1241. doi: 10.1161/01.HYP.37.5.1236, PMID: 11358934

[ref21] Lavie C. J. Arena R. Swift D. L. Johannsen N. M. Sui X. Lee D.. (2015). Exercise and the cardiovascular system: clinical science and cardiovascular outcomes. Circ. Res. 117, 207–219. doi: 10.1161/CIRCRESAHA.117.305205, PMID: 26139859 PMC4493772

[ref22] Maeda S. Iemitsu M. Miyauchi T. Kuno S. Matsuda M. Tanaka H. (2005). Aortic stiffness and aerobic exercise: mechanistic insight from microarray analyses. Med. Sci. Sports Exerc. 37, 1710–1716. doi: 10.1249/01.mss.0000175052.37087.f8, PMID: 16260970

[ref23] Manta A. Cojocaru E. Leon-Constantin M. M. Maștaleru A. Roca M. Rusu C.. (2021). IPAQ-L and CPET usefulness in a north-eastern Romanian population undergoing cardiac rehabilitation. Appl. Sci. 11:5483. doi: 10.3390/app11125483

[ref24] Mediano M. F. F. Leifer E. S. Cooper L. S. Keteyian S. J. Kraus W. E. Mentz R. J.. (2018). Influence of baseline physical activity level on exercise training response and clinical outcomes in heart failure. JACC Heart Fail. 6, 1011–1019. doi: 10.1016/j.jchf.2018.09.012, PMID: 30497641 PMC6317714

[ref25] Messner B. Bernhard D. (2014). Smoking and cardiovascular disease: mechanisms of endothelial dysfunction and early atherogenesis. Arterioscler. Thromb. Vasc. Biol. 34, 509–515. doi: 10.1161/ATVBAHA.113.300156, PMID: 24554606

[ref26] Mitchell G. F. Moyé L. A. Braunwald E. Rouleau J.-L. Bernstein V. Geltman E. M.. (1997). Sphygmomanometrically determined pulse pressure is a powerful independent predictor of recurrent events after myocardial infarction in patients with impaired left ventricular function. Circulation 96, 4254–4260. doi: 10.1161/01.CIR.96.12.4254, PMID: 9416890

[ref27] Müller J. Amberger T. Berg A. Goeder D. Remmele J. Oberhoffer R.. (2017). physical activity in adults with congenital heart disease and associations with functional outcomes. Heart 103, 1117–1121. doi: 10.1136/heartjnl-2016-310828 28274955

[ref28] Novakovic M. Jug B. Lenasi H. (2017). Clinical impact of exercise in patients with peripheral arterial disease. Vascular 25, 412–422. doi: 10.1177/1708538116678752, PMID: 28256934

[ref29] Novaković M. Prokšelj K. Starc V. Jug B. (2017). Cardiovascular autonomic dysfunction and carotid stiffness in adults with repaired tetralogy of Fallot. Clin. Auton. Res. 27, 185–192. doi: 10.1007/s10286-017-0411-0, PMID: 28275877

[ref30] Oliveira N. L. Ribeiro F. Alves A. J. Campos L. Oliveira J. (2014). The effects of exercise training on arterial stiffness in coronary artery disease patients: a state-of-the-art review. Clin. Physiol. Funct. Imaging 34, 254–262. doi: 10.1111/cpf.12093, PMID: 24138480

[ref31] Pahkala K. Heinonen O. J. Lagström H. Hakala P. Simell O. Viikari J. S. A.. (2008). Vascular endothelial function and leisure-time physical activity in adolescents. Circulation 118, 2353–2359. doi: 10.1161/CIRCULATIONAHA.108.791988, PMID: 19015403

[ref32] Payvandi L. Dyer A. McPherson D. Ades P. Stein J. Liu K.. (2009). Physical activity during daily life and brachial artery flow-mediated dilation in peripheral arterial disease. Vasc. Med. 14, 193–201. doi: 10.1177/1358863X08101018, PMID: 19651668 PMC2749502

[ref33] Pelliccia A. Sharma S. Gati S. Bäck M. Börjesson M. Caselli S.. (2020). 2020 ESC guidelines on sports cardiology and exercise in patients with cardiovascular disease. Eur. Heart J. 42, 17–96. doi: 10.1093/eurheartj/ehaa605, PMID: 32860412

[ref34] Poredoš P. (2002). Endothelial dysfunction and cardiovascular disease. Pathophysiol. Haemost. Thromb. 32, 274–277. doi: 10.1159/000073580 13679656

[ref35] Sitia S. Tomasoni L. Atzeni F. Ambrosio G. Cordiano C. Catapano A.. (2010). From endothelial dysfunction to atherosclerosis. Autoimmun. Rev. 9, 830–834. doi: 10.1016/j.autrev.2010.07.016, PMID: 20678595

[ref36] Steinberg H. O. Bayazeed B. Hook G. Johnson A. Cronin J. Baron A. D. (1997). Endothelial dysfunction is associated with cholesterol levels in the high normal range in humans. Circulation 96, 3287–3293. doi: 10.1161/01.cir.96.10.3287, PMID: 9396418

[ref37] Strath S. J. Kaminsky L. A. Ainsworth B. E. Ekelund U. Freedson P. S. Gary R. A.. (2013). Guide to the assessment of physical activity: clinical and research applications: a scientific statement from the American heart association. Circulation 128, 2259–2279. doi: 10.1161/01.cir.0000435708.67487.da, PMID: 24126387

[ref38] Tanaka H. Dinenno F. A. Monahan K. D. Clevenger C. M. DeSouza C. A. Seals D. R. (2000). Aging, habitual exercise, and dynamic arterial compliance. Circulation 102, 1270–1275. doi: 10.1161/01.CIR.102.11.1270, PMID: 10982542

[ref39] Teixeira-Lemos E. Nunes S. Teixeira F. Reis F. (2011). Regular physical exercise training assists in preventing type 2 diabetes development: focus on its antioxidant and anti-inflammatory properties. Cardiovasc. Diabetol. 10:12. doi: 10.1186/1475-2840-10-12, PMID: 21276212 PMC3041659

[ref40] Teo K. K. Ounpuu S. Hawken S. Pandey M. Valentin V. Hunt D.. (2006). Tobacco use and risk of myocardial infarction in 52 countries in the INTERHEART study: a case-control study. Lancet 368, 647–658. doi: 10.1016/S0140-6736(06)69249-0, PMID: 16920470

[ref41] Winzer E. B. Woitek F. Linke A. (2018). Physical activity in the prevention and treatment of coronary artery disease. J. Am. Heart Assoc. 7:e007725. doi: 10.1161/JAHA.117.007725, PMID: 29437600 PMC5850195

[ref42] Zieman S. J. Melenovsky V. Kass D. A. (2005). Mechanisms, pathophysiology, and therapy of arterial stiffness. Arterioscler. Thromb. Vasc. Biol. 25, 932–943. doi: 10.1161/01.ATV.0000160548.78317.29, PMID: 15731494

